# Association of B3GNT3 Expression with Tumour Stage and Lymph Node Metastasis in Colon Adenocarcinoma

**DOI:** 10.3390/ijms27146273

**Published:** 2026-07-14

**Authors:** Adam Piecuch, Jerzy Z. Piecuch, Karolina Bajdak-Rusinek, Marek Michalski, Natalia Matysiak, Marlena Brzozowa-Zasada

**Affiliations:** 1 Department of Histology and Cell Pathology in Zabrze, Faculty of Medical Sciences in Zabrze, Medical University of Silesia in Katowice, 40-055 Katowice, Poland; mmichalski@sum.edu.pl (M.M.); nmatysiak@sum.edu.pl (N.M.); mbrzozowa@sum.edu.pl (M.B.-Z.); 2 Department of General and Bariatric Surgery and Emergency Medicine in Zabrze, Faculty of Medical Sciences in Zabrze, Medical University of Silesia in Katowice, 40-055 Katowice, Poland; 3 Department of Medical Genetics, Faculty of Medical Sciences in Katowice, Medical University of Silesia in Katowice, 40-055 Katowice, Poland; kbajdak-rusinek@sum.edu.pl

**Keywords:** glycosyltransferase, glycosylation reprogramming, poly-N-acetyllactosamine, Golgi apparatus, post-transcriptional regulation, metastatic competence, immunohistochemistry

## Abstract

Aberrant glycosylation contributes to tumour progression and metastatic dissemination. Beta-1,3-N-acetylglucosaminyltransferase 3 (B3GNT3) is involved in poly-N-acetyllactosamine synthesis, but its role in colon adenocarcinoma remains insufficiently characterised. This retrospective single-centre study evaluated B3GNT3 expression and its association with clinicopathological parameters using TCGA and CPTAC data, immunohistochemistry in 97 colon adenocarcinomas, Western blot analysis, and transmission electron microscopy. TCGA analysis showed no significant differences in B3GNT3 mRNA expression between normal and tumour tissues. In contrast, CPTAC, immunohistochemistry, and Western blot analyses demonstrated increased B3GNT3 protein expression in tumour tissue compared with non-neoplastic mucosa. Immunohistochemical expression was assessed semi-quantitatively using the immunoreactive score (IRS). Lower B3GNT3 expression was associated with advanced stage and node-positive status. Because pathological stage and lymph node status are interrelated clinicopathological variables in colon adenocarcinoma, these findings were interpreted as partially overlapping associations rather than independent biological endpoints. Exploratory ROC and logistic regression analyses supported these cohort-level associations; however, the ROC-derived IRS cut-off was not externally validated, and regression models were interpreted cautiously. TEM/immunogold analysis supported Golgi/secretory-pathway localisation but was descriptive. Overall, B3GNT3 expression was associated with clinicopathological features in this retrospective cohort.

## 1. Introduction

Colorectal cancer remains one of the leading causes of cancer-related morbidity and mortality worldwide, with colon adenocarcinoma accounting for the majority of cases [1,2]. Despite substantial advances in molecular classification and systemic therapies, pathological staging based on the TNM system remains the cornerstone for prognostic assessment and therapeutic decision-making in routine clinical practice [3]. Nevertheless, considerable heterogeneity in clinical outcomes is observed among patients within the same TNM categories, particularly regarding lymph node involvement and progression to advanced-stage disease [4,5]. This variability highlights the need to investigate additional molecular and protein-expression features that may be associated with conventional clinicopathological parameters. In this context, immunohistochemistry (IHC) enables direct assessment of protein expression within preserved tissue architecture and remains widely applicable in routine pathological practice. In contrast to transcriptomic analyses, protein-level evaluation may better reflect biologically relevant alterations occurring during tumour progression [6,7,8]. This distinction is particularly important in glycosylation-related pathways, where post-transcriptional regulation may substantially influence protein abundance and localization.

Aberrant glycosylation is increasingly recognised as a hallmark of malignant transformation and tumour progression [9,10]. Altered glycosyltransferase activity affects cell adhesion, receptor signalling, immune recognition, loss of epithelial differentiation, and clinicopathological heterogeneity [10,11]. Among enzymes involved in the synthesis of poly-N-acetyllactosamine chains, beta-1,3-N-acetylglucosaminyltransferase 3 (B3GNT3) has attracted increasing attention because of its role in glycan elongation and regulation of cell surface glycoproteins [12]. Dysregulation of glycosyltransferases has been implicated in tumour invasion and metastatic dissemination in several malignancies, including cervical cancer, lung adenocarcinoma, and hepatocellular carcinoma [13,14,15]. However, the clinicopathological significance of B3GNT3 in colon adenocarcinoma remains insufficiently characterised.

Publicly available transcriptomic and proteomic datasets provide valuable insights into molecular alterations associated with tumour progression. However, discrepancies frequently occur between mRNA and protein expression levels due to post-transcriptional regulation, protein degradation, translational control, and tumour microenvironmental influences [16,17]. Therefore, validation of candidate biomarkers at the protein level within clinically characterised patient cohorts remains essential. It should be pointed out that in the absence of survival outcomes, treatment-response data, or external validation, such analyses should be interpreted as assessments of clinicopathological association rather than evidence of clinical utility. 

In the present study, we investigated B3GNT3 expression in colon adenocarcinoma using an integrative approach combining publicly available transcriptomic datasets, proteomic analyses, and immunohistochemical evaluation in an independent clinical cohort. Our primary objective was to determine whether B3GNT3 protein expression is associated with advanced-stage disease and lymph node metastasis. Furthermore, we evaluated whether the incorporation of B3GNT3 expression into logistic regression models provides incremental contribution to model fit beyond conventional pathological T (pT) classification. This study was designed to assess clinicopathological associations, not to establish B3GNT3 as a clinically validated prognostic or predictive biomarker. Advanced-stage disease and lymph node metastasis were therefore interpreted as overlapping, clinically related outcomes rather than independent biological endpoints.

## 2. Results

### 2.1. mRNA B3GNT3 Expression in COAD Based on TCGA Transcriptomic Data

**Figure 1:**
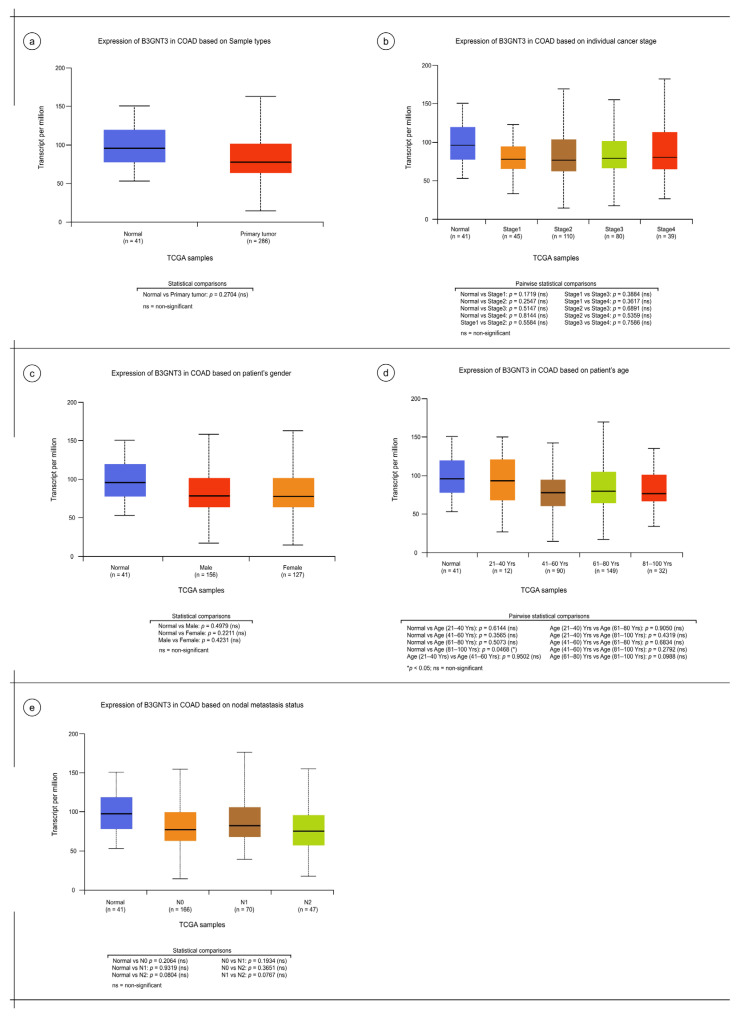
B3GNT3 mRNA expression in colon adenocarcinoma based on TCGA data accessed through the UALCAN platform. B3GNT3 transcript levels are presented according to sample type (normal colon tissue vs. primary tumour) (**a**), pathological tumour stage (I–IV) (**b**), patient sex (**c**), patient age (**d**), and lymph node metastasis status (N0–N2) (**e**). Pairwise statistical comparisons are shown directly below each panel. B3GNT3 mRNA expression did not differ significantly between normal colon tissue and primary tumour samples ((**a**), *p* = 0.2704), and no consistent statistically significant differences were observed across pathological stage, sex, age, or nodal-status subgroups. Boxes represent interquartile ranges, horizontal lines indicate median values, and whiskers indicate minimum–maximum ranges. ns, non-significant; TCGA, The Cancer Genome Atlas; COAD, colon adenocarcinoma.

B3GNT3 mRNA expression was analysed in colon adenocarcinoma (COAD) samples from The Cancer Genome Atlas (TCGA) using the UALCAN platform. Transcript levels were detectable in both normal colonic mucosa and tumour tissues, with comparable expression observed across pathological stages, sex subgroups, and lymph node categories (Figure 1a–e). No statistically significant differences in B3GNT3 mRNA expression were identified between normal and tumour tissues or among clinicopathological subgroups (all *p* > 0.05), although a non-significant trend toward lower expression was observed in N2 tumours. Overall, transcriptomic analysis did not demonstrate a significant association between B3GNT3 mRNA expression and tumour progression parameters in COAD.

### 2.2. B3GNT3 Protein Expression in COAD Based on CPTAC Data

**Figure 2:**
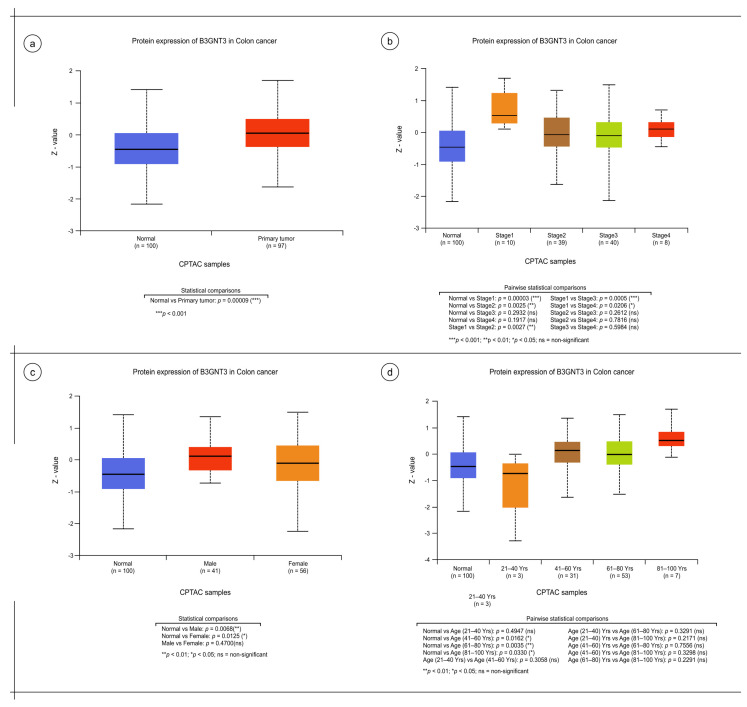
B3GNT3 protein expression in colon adenocarcinoma based on CPTAC data accessed through the UALCAN platform. B3GNT3 protein abundance is presented as Z-values according to sample type (normal colon tissue vs. primary tumour) (**a**), pathological tumour stage (I–IV) (**b**), patient sex (**c**), and patient age (**d**). Pairwise statistical comparisons are shown directly below each panel. B3GNT3 protein expression was significantly higher in primary tumour samples compared with normal colon tissue ((**a**), *p* = 0.00009). Stage-stratified analysis showed a stage-dependent distribution, with the most pronounced differences observed in early-stage tumours; significant and non-significant pairwise comparisons are indicated below panel (**b**). In sex-stratified analyses, the normal tissue group was used as the available CPTAC reference normal cohort for comparison with male and female tumour subgroups. Boxes represent interquartile ranges, horizontal lines indicate median values, and whiskers indicate minimum–maximum ranges. ns, non-significant; CPTAC, Clinical Proteomic Tumor Analysis Consortium.

B3GNT3 protein expression was analysed using CPTAC colon adenocarcinoma data accessed through the UALCAN platform. In contrast to transcriptomic findings, B3GNT3 protein levels were increased in tumour tissues compared with normal colonic mucosa and demonstrated a stage-dependent distribution (Figure 2a–d). The highest protein expression was observed in Stage I tumours, which showed significantly higher B3GNT3 levels than normal tissues as well as Stage II–IV tumours (all *p* < 0.05). Protein expression remained elevated in Stage II tumours compared with normal tissue, whereas no significant differences were detected among Stage II, III, and IV cancers. Sex-stratified analysis demonstrated significantly higher B3GNT3 protein expression in tumour tissues than in normal colonic samples in both male and female patients, without significant differences between sexes. Overall, these findings indicate that B3GNT3 protein expression, unlike mRNA expression, is increased in colon adenocarcinoma, particularly in early-stage disease.

### 2.3. Immunohistochemical Analysis of B3GNT3 Expression in Colon Adenocarcinoma

#### 2.3.1. Clinicopathological Characteristics of the Study Cohort

The study cohort included 97 patients with histologically confirmed colon adenocarcinoma. Detailed clinicopathological characteristics are summarised in Table 1. There were 48 women (49.48%) and 49 men (50.52%). The mean age at diagnosis was 67.8 ± 12.3 years, with a median of 68 years and a range of 33–89 years.

According to the AJCC TNM classification, 10 patients (10.31%) had Stage I disease, 37 (38.14%) had Stage II, 43 (44.33%) had Stage III, and 7 (7.22%) had Stage IV disease. Early-stage disease (Stage I–II) was present in 47 patients (48.45%), while advanced-stage disease (Stage III–IV) was observed in 50 patients (51.55%).

Histological grading revealed 14 well-differentiated tumours (G1; 14.43%), 59 moderately differentiated tumours (G2; 60.82%), and 24 poorly differentiated tumours (G3; 24.74%). Most tumours were classified as pT3 (68.04%) or pT4 (18.56%). Lymph node metastases (pN1–2) were identified in 48 patients (49.48%). Left-sided tumours were slightly more frequent than right-sided tumours (55.67% vs. 44.33%).

#### 2.3.2. B3GNT3 Immunohistochemical Expression and Clinicopathological Parameters

**Figure 3:**
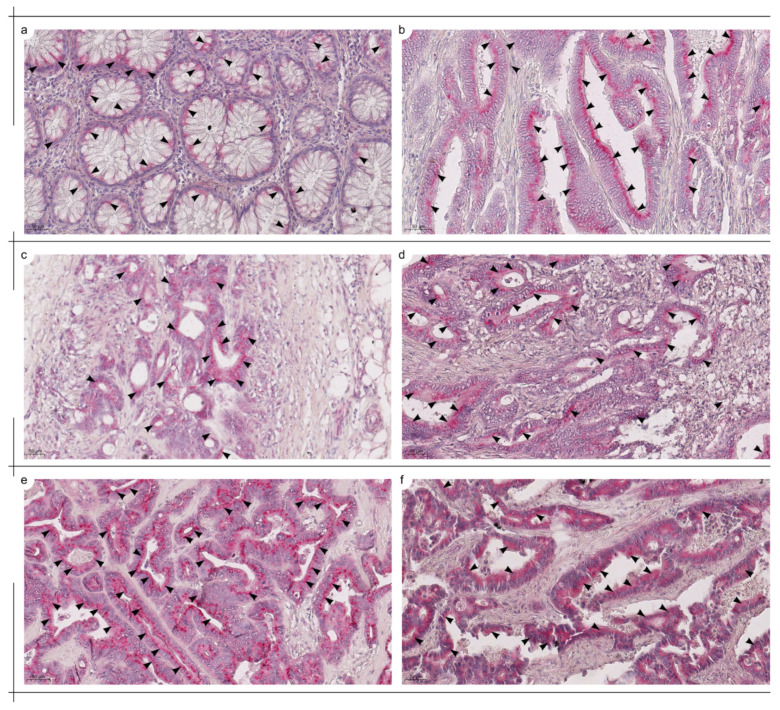
Representative immunohistochemical expression of B3GNT3 in non-neoplastic colonic mucosa and colon adenocarcinoma tissues. B3GNT3 immunoreactivity is visualised as red cytoplasmic and/or apical cytoplasmic staining, whereas nuclei are counterstained with haematoxylin and appear blue–purple. Non-neoplastic colonic mucosa shows detectable B3GNT3 expression in glandular epithelial cells (**a**). Colon adenocarcinoma specimens demonstrate heterogeneous B3GNT3 expression within neoplastic glands and tumour cell clusters (**b**–**f**). Arrowheads indicate representative B3GNT3-positive epithelial or tumour cells. Scale bars: 50 µm. Representative negative-control sections processed by omission of the primary antibody are provided in Supplementary Figure S1 and showed no relevant nonspecific staining.

Immunohistochemical analysis demonstrated heterogeneous B3GNT3 expression across colon adenocarcinoma samples (Figure 3).

**Figure 4:**
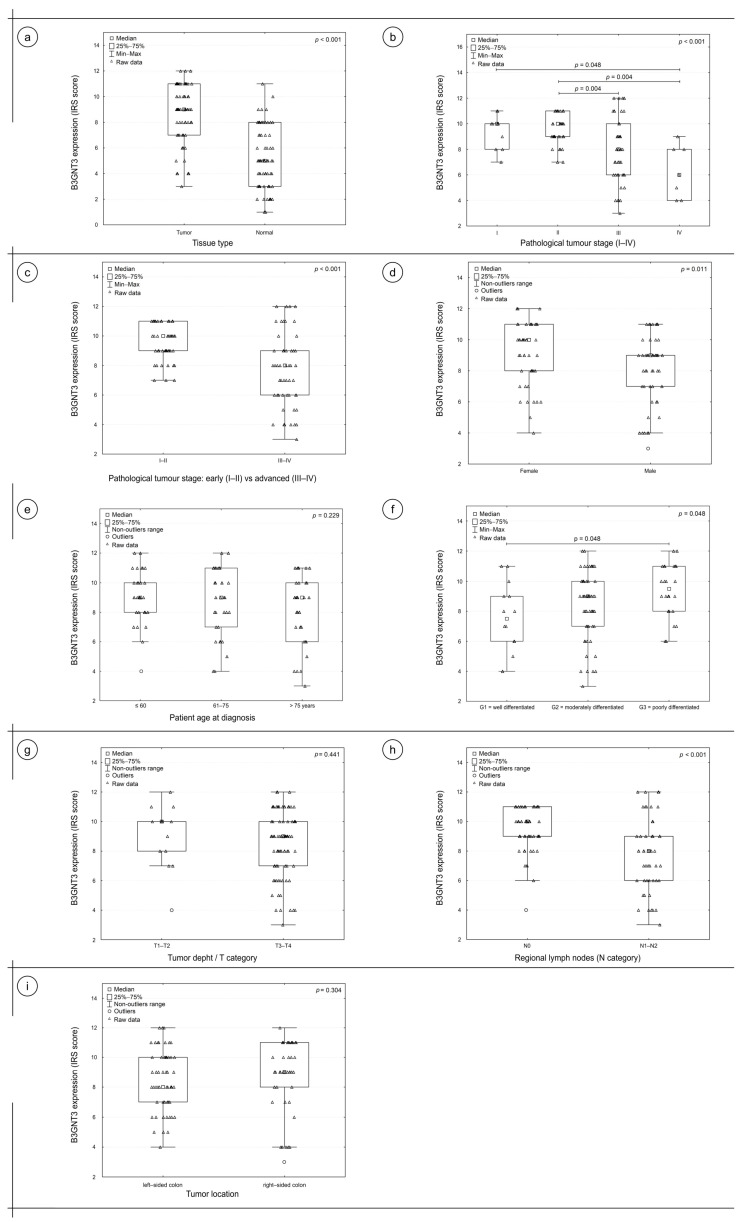
B3GNT3 immunohistochemical expression in colon adenocarcinoma and its association with clinicopathological parameters. B3GNT3 expression was assessed semi-quantitatively using the immunoreactive score (IRS). Individual patient-level IRS values are shown as raw data points overlaid on box-and-whisker plots to visualise data distribution and potential outliers. B3GNT3 expression was significantly higher in tumour tissue compared with adjacent non-neoplastic mucosa ((**a**), *p* < 0.001). Expression differed significantly across pathological tumour stages (I–IV), with significant pairwise post hoc comparisons indicated directly on the plot (**b**). B3GNT3 expression was higher in early-stage tumours (Stage I–II) than in advanced-stage tumours (Stage III–IV) ((**c**), *p* < 0.001). Female patients showed higher B3GNT3 expression than male patients ((**d**), *p* = 0.011). No significant association was observed with patient age at diagnosis ((**e**), *p* = 0.229), tumour invasion depth/T category ((**g**), *p* = 0.441), or tumour location ((**i**), *p* = 0.304). B3GNT3 expression differed according to histological grade, with significant pairwise comparison indicated on the plot (**f**). Lower B3GNT3 expression was observed in node-positive tumours (N1–N2) compared with node-negative tumours (N0) ((**h**), *p* < 0.001). Because pathological stage and lymph node status are interrelated clinicopathological variables in colon cancer, panels (**b**,**c**,**h**) should be interpreted as complementary analyses of related clinicopathological features rather than independent biological endpoints. Boxes represent the interquartile range (25–75%), horizontal lines indicate median values, squares indicate mean values, whiskers represent minimum–maximum or non-outlier ranges, and circles indicate outliers. *p*-values and significant pairwise comparisons are indicated directly on the plots.

Comparative analysis between tumour tissue and corresponding non-neoplastic mucosa demonstrated significantly higher B3GNT3 expression in tumour samples (Table 2; Figure 4a). The mean IRS in tumour tissue was 8.66 ± 2.28, with a median of nine and a range of 3–12. In non-neoplastic mucosa, the mean IRS was 5.29 ± 2.39, with a median of five and a range of 1–11. This difference was statistically significant (Z = 6.870, *p* < 0.001). B3GNT3 expression was quantified using the IRS scale (0–12) in the analysed cohort of 97 colon adenocarcinoma cases, and the resulting numerical IRS values were used for all statistical comparisons presented in Table 2 and Figure 4.

When analysed according to pathological stage, B3GNT3 expression demonstrated a significant stage-dependent pattern (H = 19.642, *p* < 0.001) (Table 2; Figure 4b). Median expression was highest in Stage I and Stage II tumours, both with median IRS values of 10, and lower in Stage III and Stage IV tumours, with median values of eight and six, respectively. Post hoc analysis confirmed significantly higher expression in Stage I compared with Stage IV tumours and in Stage II compared with Stage III and Stage IV tumours.

A significant negative correlation was observed between B3GNT3 expression and tumour stage (R = −0.425, *p* < 0.001). When stages were grouped, B3GNT3 expression remained significantly higher in early-stage tumours (Stage I–II) than in advanced-stage tumours (Stage III–IV) (Z = 4.107, *p* < 0.001) (Figure 4c).

Analysis according to lymph node status demonstrated significantly lower B3GNT3 expression in node-positive tumours (pN1–2) compared with node-negative tumours (pN0) (Z = 3.529, *p* < 0.001) (Table 2; Figure 4h).

Because AJCC stage III colon cancer is largely defined by the presence of regional lymph node metastasis, pathological stage and nodal status are interrelated clinicopathological variables. Accordingly, the associations observed for advanced stage and lymph node metastasis should be interpreted as complementary and partially overlapping findings, rather than as evidence for two independent biological endpoints.

A statistically significant association was also observed between B3GNT3 expression and histological grade (H = 6.055, *p* = 0.048), with higher expression in poorly differentiated tumours compared with well-differentiated tumours (Table 2; Figure 4f). However, this association was weaker than those observed for stage and lymph node status.

B3GNT3 expression was significantly higher in tumours from female patients than in tumours from male patients (Z = 2.551, *p* = 0.011) (Figure 4d). No significant associations were found between B3GNT3 expression and patient age, tumour invasion depth, or tumour location (Table 2; Figure 4e,g).

### 2.4. Exploratory ROC Analysis of B3GNT3 Expression and ROC-Derived Cut-Off

**Figure 5:**
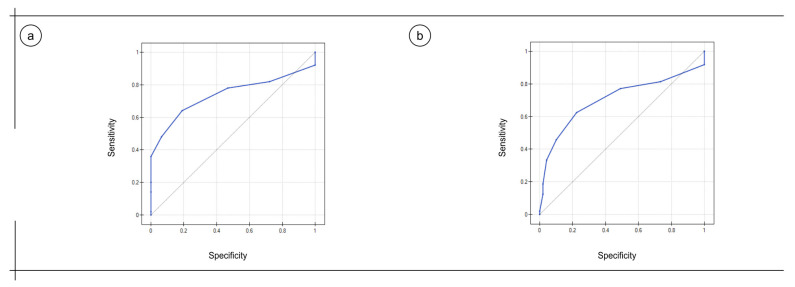
Exploratory receiver operating characteristic (ROC) curve analysis of B3GNT3 protein expression in colon adenocarcinoma. ROC analysis was performed to assess cohort-level separation between early- and advanced-stage disease (Stage III–IV vs. Stage I–II) (**a**) and between node-positive and node-negative tumours (N1–N2 vs. N0) (**b**). The ROC-derived IRS cut-off value of 8 was identified and evaluated in the same retrospective cohort and was therefore treated as exploratory. The diagonal grey line represents the reference line of no discrimination (AUC = 0.5), whereas the blue curves illustrate the observed separation based on B3GNT3 expression.

ROC curve analysis was performed to evaluate the discriminatory performance of B3GNT3 expression for advanced-stage disease and lymph node metastasis (Figure 5; Table 3). The ROC-derived IRS cut-off value of eight was identified using the Youden index in the same retrospective dataset in which its performance was estimated; therefore, this threshold should be regarded as exploratory and hypothesis-generating rather than validated. No independent validation cohort was available, and no separate training–validation split was performed because of the limited cohort size. Consequently, the reported AUC values, sensitivity, specificity, positive predictive value (PPV), and negative predictive value (NPV) may overestimate performance and should not be interpreted as evidence of diagnostic, prognostic, or predictive clinical utility.

For advanced-stage disease (Stage III–IV vs. Stage I–II), B3GNT3 expression demonstrated moderate discriminatory ability, with an AUC of 0.740 (95% CI: 0.636–0.843; *p* < 0.001). Using the exploratory IRS cut-off of eight, sensitivity was 64.00% (95% CI: 50.14–75.86), specificity was 80.85% (95% CI: 67.46–89.58), PPV was 78.05% (95% CI: 63.29–88.00), and NPV was 67.86% (95% CI: 54.82–78.60). For lymph node metastasis (pN1–2 vs. pN0), B3GNT3 expression also showed only moderate separation, with an AUC of 0.706 (95% CI: 0.597–0.814; *p* < 0.001). At the same exploratory IRS cut-off of eight, sensitivity was 62.50% (95% CI: 48.36–74.78), specificity was 77.55% (95% CI: 64.12–86.98), PPV was 73.17% (95% CI: 58.07–84.31), and NPV was 67.86% (95% CI: 54.82–78.60).

These results indicate that B3GNT3 expression was associated with advanced-stage and node-positive status in this cohort; however, the moderate AUC values and the absence of independent cut-off validation require cautious interpretation. The cut-off value of 8 was therefore used only for exploratory dichotomisation in subsequent models and should not be considered a clinically established threshold.

### 2.5. Logistic Regression Analysis for Advanced Clinical Stage

Logistic regression analysis was performed to identify factors associated with advanced clinical stage (Stage III–IV vs. Stage I–II). The analysed variables included sex, age, histological grade, tumour invasion depth, tumour location, and B3GNT3 expression. Because nodal status contributes directly to AJCC stage classification, pN status was not entered as an adjustment variable in models with advanced stage as the outcome, in order to avoid circular adjustment for a component of the endpoint. These models should be interpreted as exploratory analyses of association with stage grouping. For this endpoint, 50 events were available; with six predictors included in the full multivariable models, the events-per-variable ratio was 8.3. This value is below the conventional threshold of 10 events per variable, and the number of Stage IV cases was small (n = 7); therefore, the full regression models should be interpreted cautiously and as exploratory.

#### 2.5.1. B3GNT3 as a Continuous Variable

In univariate analysis, B3GNT3 expression was significantly associated with advanced-stage disease. Higher B3GNT3 expression was associated with a lower likelihood of advanced disease (OR = 0.608; 95% CI: 0.474–0.779; *p* < 0.001) (Table 4). Tumour invasion depth was also significantly associated with advanced stage, with T3/T4 tumours showing increased odds of advanced disease compared with T1/T2 tumours (OR = 4.234; 95% CI: 1.086–16.501; *p* = 0.038). In the multivariate model, B3GNT3 expression remained associated with advanced-stage disease after adjustment for selected clinicopathological variables (OR = 0.607; 95% CI: 0.464–0.794; *p* < 0.001). Tumour invasion depth showed borderline statistical significance (OR = 4.760; 95% CI: 0.973–23.296; *p* = 0.054), whereas sex, age, histological grade, and tumour location were not significantly associated with advanced stage (Table 4).

#### 2.5.2. B3GNT3 as a Dichotomous Variable

B3GNT3 expression was also analysed as a dichotomous variable using the exploratory ROC-derived cut-off value of 8. Because this cut-off was derived and evaluated in the same dataset, the dichotomised analysis was considered exploratory and was not used to define a validated clinical threshold.

In univariate analysis, low B3GNT3 expression (≤8) was strongly associated with advanced-stage disease compared with higher expression (>8) (OR = 7.506; 95% CI: 2.967–18.988; *p* < 0.001) (Table 5).

In the multivariate model, low B3GNT3 expression remained associated with advanced-stage disease after adjustment for selected clinicopathological variables (OR = 8.605; 95% CI: 3.001–24.673; *p* < 0.001). Tumours classified as T3/T4 were also associated with advanced stage (OR = 5.320; 95% CI: 1.073–26.371; *p* = 0.041). No significant associations were observed for sex, age, histological grade, or tumour location (Table 5).

### 2.6. Incremental Association of B3GNT3 with Advanced Stage in Exploratory Models

To assess whether B3GNT3 expression adds information on model fit beyond tumour invasion depth, reduced logistic regression models including only pT were compared with extended exploratory models including both pT and B3GNT3 expression. This analysis was restricted to pT-based models and does not imply independence from nodal status, because nodal involvement is incorporated into AJCC stage classification.

When B3GNT3 was analysed as a continuous variable, the reduced model including only pT showed limited explanatory power, with a pseudo-R^2^ of 0.038. After the addition of B3GNT3 expression, pseudo-R^2^ increased to 0.188. Model comparison confirmed a significant improvement in fit after inclusion of B3GNT3 (*p* < 0.001) (Table 6).

When B3GNT3 was analysed as a dichotomous variable, the addition of low B3GNT3 expression to the pT-only model increased pseudo-R^2^ from 0.038 to 0.198. The improvement in model fit was also statistically significant (*p* < 0.001) (Table 7).

These findings indicate that B3GNT3 expression adds cohort-level clinicopathological information associated with advanced-stage disease beyond tumour invasion depth alone. They do not demonstrate clinical predictive utility.

### 2.7. Logistic Regression Analysis for Lymph Node Metastasis

Logistic regression analysis was performed to identify factors associated with lymph node metastasis (pN1–2 vs. pN0). This endpoint was analysed as a clinically important component of stage classification and as a complementary analysis to the advanced-stage endpoint, not as a fully independent biological outcome. For this endpoint, 48 events were available; with six predictors included in the full multivariable models, the events-per-variable ratio was 8.0. This again falls below the conventional threshold of 10 events per variable, supporting cautious interpretation of the multivariable estimates.

#### 2.7.1. B3GNT3 as a Continuous Variable

In univariate analysis, higher B3GNT3 expression was associated with a lower likelihood of lymph node metastasis (OR = 0.677; 95% CI: 0.542–0.846; *p* < 0.001) (Table 8). Tumour invasion depth was also significantly associated with nodal involvement, with T3/T4 tumours showing increased odds of lymph node metastasis compared with T1/T2 tumours (OR = 6.658; 95% CI: 1.390–31.897; *p* = 0.018).

In the multivariate model, B3GNT3 expression remained associated with lymph node metastasis after adjustment for selected clinicopathological variables (OR = 0.680; 95% CI: 0.532–0.868; *p* = 0.002). Tumour invasion depth also remained significant (OR = 7.868; 95% CI: 1.437–43.098; *p* = 0.017), whereas sex, age, histological grade, and tumour location were not significantly associated with nodal status (Table 8).

#### 2.7.2. B3GNT3 as a Dichotomous Variable

Using the ROC-derived cut-off value of eight, low B3GNT3 expression (≤8) was strongly associated with lymph node metastasis in univariate analysis (OR = 5.758; 95% CI: 2.365–14.018; *p* < 0.001) (Table 9). This dichotomisation should be interpreted cautiously because the threshold was not validated in an independent cohort.

In the multivariate model, low B3GNT3 expression remained associated with lymph node metastasis after adjustment for selected clinicopathological variables (OR = 6.216; 95% CI: 2.262–17.081; *p* < 0.001). Tumour invasion depth was also associated with nodal involvement (OR = 8.769; 95% CI: 1.551–49.565; *p* = 0.014). No significant associations were observed for sex, age, histological grade, or tumour location (Table 9).

### 2.8. Incremental Association of B3GNT3 with Lymph Node Metastasis in Exploratory Models

Reduced logistic regression models, including only pT, were compared with extended exploratory models, including both pT and B3GNT3 expression, to evaluate whether B3GNT3 added information on model fit for lymph node metastasis in this cohort. These models were considered complementary to, and partially overlapping with, analyses of the advanced stage. 

When B3GNT3 was analysed as a continuous variable, the pT-only model showed a pseudo-R^2^ of 0.057. After the addition of B3GNT3 expression, pseudo-R^2^ increased to 0.184. Model comparison demonstrated a significant improvement in fit (*p* < 0.001) (Table 10).

When B3GNT3 was analysed as a dichotomous variable, inclusion of low B3GNT3 expression increased pseudo-R^2^ from 0.057 to 0.159. The improvement in model fit was statistically significant (*p* < 0.001) (Table 10).

These findings suggest that B3GNT3 expression provides additional cohort-level information associated with lymph node involvement beyond tumour invasion depth. They should not be interpreted as demonstrating predictive clinical performance.

### 2.9. Events-per-Variable Assessment, Model Diagnostics, and Internal Validation of Exploratory Regression Models

Given the limited cohort size, additional analyses were performed to assess potential overfitting and model instability in the exploratory multivariable logistic regression models. The full models included six predictors: sex, age, B3GNT3 expression, histological grade, tumour invasion depth, and tumour location. For the advanced-stage endpoint, 50 events were available, corresponding to an events-per-variable ratio (EPV) of 8.3. For the lymph-node-metastasis endpoint, 48 events were available, corresponding to an EPV of 8.0. Both values are below the conventional threshold of 10 events per variable, indicating that the full multivariable models may be vulnerable to overfitting.

Multicollinearity diagnostics did not indicate substantial collinearity among predictors. The maximum variance inflation factor was 1.18 for models including B3GNT3 as a continuous variable and 1.13 for models including dichotomised B3GNT3 expression. Calibration was assessed using the Hosmer–Lemeshow goodness-of-fit test and Brier scores. The Hosmer–Lemeshow test did not indicate a significant lack of fit for the advanced-stage model with continuous B3GNT3 (*p* = 0.130), the advanced-stage model with dichotomised B3GNT3 (*p* = 0.979), the lymph-node-metastasis model with continuous B3GNT3 (*p* = 0.191), or the lymph-node-metastasis model with dichotomised B3GNT3 (*p* = 0.901). The corresponding apparent Brier scores were 0.188, 0.183, 0.196, and 0.188, respectively.

Internal validation was performed using bootstrap resampling and repeated 10-fold cross-validation. Bootstrap optimism correction reduced the apparent AUC from 0.778 to 0.725 for the advanced-stage model with continuous B3GNT3 and from 0.789 to 0.739 for the model with dichotomised B3GNT3. For lymph node metastasis, the corresponding optimism-corrected AUC values were 0.710 and 0.726. Repeated 10-fold cross-validation yielded mean AUC values of 0.720 and 0.696 for the advanced-stage models and 0.703 and 0.675 for the lymph-node-metastasis models. These findings indicate modest optimism and support a cautious interpretation of the regression models. Accordingly, the regression results are presented as exploratory associations rather than as stable clinical prediction models.

The wide confidence intervals observed for several odds ratios, particularly those involving tumour invasion depth, were retained in the Results and interpreted as evidence of limited precision and possible model instability.

### 2.10. Western Blot Validation of B3GNT3 Protein Expression

**Figure 6:**
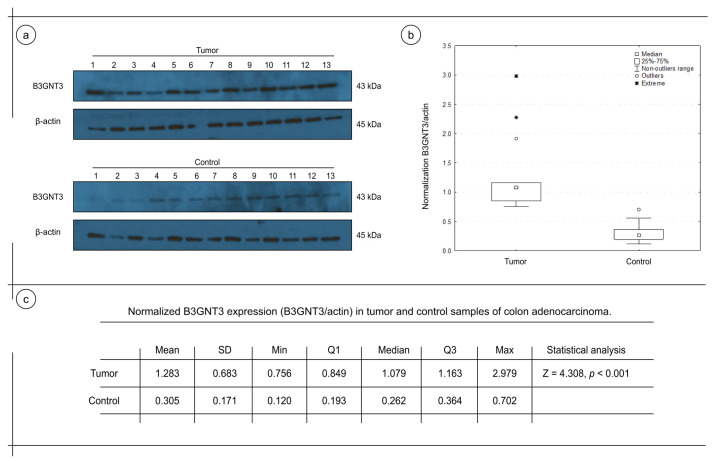
Western blot validation and quantitative analysis of B3GNT3 protein expression in colon adenocarcinoma. Representative Western blot images demonstrating B3GNT3 protein expression in tumour and corresponding paired control tissues are shown in panel (**a**). B3GNT3 protein was detected at approximately 43 kDa, whereas β-actin (~45 kDa) was used as a loading control. Quantitative densitometric analysis of normalised B3GNT3 expression (B3GNT3/β-actin ratio) in tumour and paired control samples is presented as a box-and-whisker plot in panel (**b**). Boxes represent the interquartile range (25–75%), horizontal lines indicate median values, whiskers represent non-outlier ranges, circles indicate outliers, and asterisks indicate extreme values. Summary statistics of normalised B3GNT3 expression are presented in panel (**c**). Densitometric analysis performed in 13 paired tumour and control samples demonstrated significantly higher B3GNT3 expression in tumour tissues compared with paired non-neoplastic controls (paired analysis: Z = 4.308, *p* < 0.001). Tumour and corresponding control samples were resolved on separate gels because of the total number of lanes required; however, all membranes were processed in parallel under identical experimental conditions, including transfer, blocking, antibody incubation, detection, and exposure. Densitometric values were normalised to β-actin.

Western blot analysis was performed in a subset of tumour and control samples to validate B3GNT3 protein-level differences observed by immunohistochemistry (Figure 6).

B3GNT3 protein expression was significantly higher in tumour tissue than in non-neoplastic control tissue. Mean normalised expression was 1.283 ± 0.683 in tumour samples and 0.305 ± 0.171 in controls (Z = 4.308, *p* < 0.001) (Figure 6b,c).

Tumour samples also demonstrated greater inter-sample variability, indicating marked inter-tumoural heterogeneity. Although the Western blot analysis was performed in a limited subset of samples, these findings support the protein-level upregulation of B3GNT3 observed in immunohistochemical analysis.

### 2.11. Subcellular Localisation of B3GNT3

Immunogold labelling by the use of TEM was performed as a supportive ultrastructural localisation study to assess the subcellular distribution of B3GNT3 immunoreactivity in colon adenocarcinoma tissue and was intended to demonstrate the presence and intracellular localisation of B3GNT3 labelling rather than to quantify B3GNT3 expression levels.

**Figure 7:**
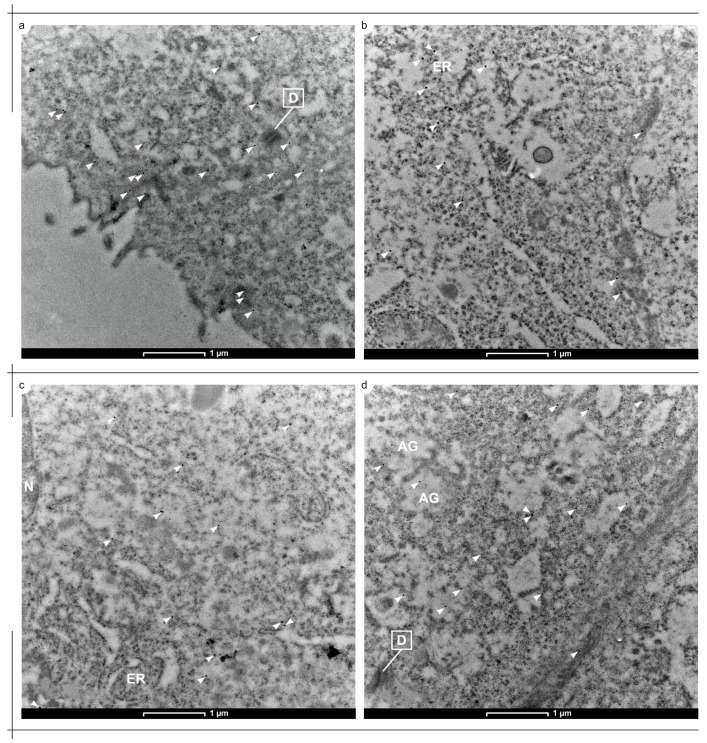
Ultrastructural localisation of B3GNT3 in normal colonocytes and colon adenocarcinoma cells. Representative transmission electron microscopy (TEM) images demonstrate the subcellular distribution of electron-dense immunogold-labelled deposits (arrowheads) in normal colonocytes (**a**) and colon adenocarcinoma cells (**b**–**d**). Immunogold particles are observed within the cytoplasm and in close association with intracellular membranous organelles, including the endoplasmic reticulum (ER) and Golgi apparatus (AG), consistent with localisation within the secretory pathway. D indicates desmosomes. Scale bars: 1 µm. Images are representative qualitative localisation examples only; immunogold particle density was not quantified, and no statistical comparison of TEM labelling intensity was performed.

B3GNT3 immunogold labelling was predominantly observed in the supranuclear cytoplasm and in close association with membranous organelles compatible with the Golgi apparatus and components of the secretory pathway. Additional labelling was occasionally observed near rough endoplasmic reticulum membranes and in the perinuclear cytoplasm (Figure 7). These findings are consistent with the expected intracellular localisation of a glycosyltransferase involved in glycoprotein processing.

## 3. Discussion

The present study demonstrated significant alterations in B3GNT3 expression in colon adenocarcinoma and supports the concept that dysregulated glycosylation represents an important component of colorectal carcinogenesis. Aberrant glycosylation is increasingly recognised as a hallmark of malignant transformation and contributes to tumour progression through modulation of cell adhesion, migration, receptor signalling, immune interactions, and epithelial plasticity [9,10]. Within this context, glycosyltransferases have emerged as biologically relevant mediators of tumour behaviour. In the present study, B3GNT3 was evaluated as a protein-expression feature associated with clinicopathological parameters rather than as a clinically validated prognostic or predictive biomarker.

B3GNT3 belongs to the β-1,3-N-acetylglucosaminyltransferase family and participates in the synthesis of poly-N-acetyllactosamine chains involved in glycoprotein maturation and cell surface interactions [12]. Glycosylation-dependent modification of membrane proteins and adhesion molecules has been implicated in tumour invasion, metastatic dissemination, and epithelial–mesenchymal transition (EMT) [16,18]. Experimental studies further demonstrated that altered B3GNT3 activity may influence tumour cell motility and invasive potential through modulation of glycan-mediated signalling pathways [16].

One of the most important findings of the present study was the discrepancy observed between transcriptomic and protein-level expression patterns. TCGA-based analyses did not demonstrate significant differences in B3GNT3 mRNA expression between normal and tumour tissues or across clinicopathological subgroups. In contrast, protein-level analyses indicated altered B3GNT3 expression in colon adenocarcinoma tissue. CPTAC analysis showed increased B3GNT3 protein expression in primary tumours compared with normal tissue, with the most pronounced differences observed in early-stage tumours, whereas immunohistochemistry and Western blot analyses demonstrated increased B3GNT3 protein expression in tumour tissue compared with non-neoplastic mucosa. Moreover, protein expression showed a non-linear stage-associated pattern, with the highest expression observed in early-stage tumours and lower expression in advanced-stage disease.

This discordance between mRNA and protein findings is biologically relevant and likely reflects the complex post-transcriptional regulation of glycosyltransferases in cancer biology. Increasing evidence indicates that mRNA abundance does not necessarily correlate directly with protein expression, particularly for enzymes involved in post-translational modification pathways [19]. Translational regulation, altered protein degradation, microRNA-mediated control, and tumour microenvironmental influences may substantially affect final protein abundance independently of transcript levels. Therefore, the present findings highlight the importance of protein-based pathological assessment and suggest that immunohistochemistry may better reflect the functional biological activity of B3GNT3 within tumour tissue than transcriptomic analyses alone.

Importantly, immunohistochemical analysis demonstrated significantly increased B3GNT3 expression in tumour tissue compared with adjacent non-neoplastic mucosa. These findings indicate that altered B3GNT3 expression represents a feature of malignant transformation in colon adenocarcinoma. Similar observations have been reported in several other malignancies. Zhang et al. demonstrated that elevated B3GNT3 expression in cervical cancer was associated with tumour progression and poor clinical outcome [20]. Likewise, increased B3GNT3 expression has been observed in non-small cell lung cancer and lung adenocarcinoma, where it correlated with aggressive tumour behaviour and adverse prognosis [11,15].

However, the present study also revealed findings that differ substantially from those previously reported in several other tumour types. In our cohort, reduced B3GNT3 protein expression was associated with advanced stage and nodal status in adjusted exploratory models. with advanced-stage disease and lymph node metastasis. In contrast, studies in cervical cancer and lung cancer demonstrated that increased B3GNT3 expression correlated with advanced TNM stage, lymph node metastasis, and poor prognosis [11,15,20]. Wu et al. additionally reported associations between high B3GNT3 expression, enhanced immune infiltration, and unfavourable survival in lung adenocarcinoma [15], whereas the current study did not demonstrate a clear relationship between B3GNT3 expression and inflammatory infiltration in colon adenocarcinoma. These discrepancies are particularly interesting and likely reflect tumour-specific differences in glycosylation biology. Glycosylation is a highly dynamic process that changes substantially during tumour evolution and may exert context-dependent biological effects depending on tissue origin, tumour microenvironment, and molecular background [9,10,21]. In some malignancies, persistent B3GNT3 overexpression may promote metastatic dissemination and tumour progression. In contrast, in colon adenocarcinoma, the observed expression pattern may suggest that B3GNT3 participates differently across stages of tumour progression associated with malignant transformation and epithelial dedifferentiation, whereas progression toward metastatic disease may involve broader alterations in glycosylation-related pathways accompanied by partial loss of specific glycosyltransferase activity. This interpretation is supported by the stage-dependent decline in B3GNT3 expression observed in the present study. This interpretation is supported by the stage-associated pattern of B3GNT3 expression observed in the present study. The highest expression levels were identified in Stage I–II tumours, whereas lower expression was observed in Stage III–IV disease and in node-positive tumours. These findings suggest that B3GNT3 expression is associated with clinicopathological stage and nodal status in this cohort. However, because pathological stage and nodal status are interrelated clinicopathological variables in colon cancer, these associations should be interpreted as partially overlapping findings rather than independent biological endpoints. Importantly, because survival, recurrence, treatment-response, and external validation data were not available, these findings do not establish prognostic or predictive value.

Interestingly, despite lower expression in advanced-stage and node-positive tumours, B3GNT3 expression remained significantly associated with histological grade, with higher expression observed in poorly differentiated lesions. Although this association was weaker than those observed for stage and nodal status, it suggests that B3GNT3 may still participate in biological processes related to tumour dedifferentiation and epithelial plasticity. Histological grade primarily reflects intrinsic tumour biology and loss of epithelial organisation, whereas TNM stage reflects anatomical tumour dissemination. Therefore, the coexistence of increased expression in poorly differentiated tumours and decreased expression in advanced-stage disease likely reflects the complex and non-linear nature of glycosylation remodelling during colorectal cancer progression.

Another important observation was the lack of a significant association between B3GNT3 expression and tumour invasion depth (pT). In contrast, strong associations were identified with lymph node metastasis and overall clinical stage. These findings may indicate that B3GNT3-related glycosylation alterations are more closely associated with nodal dissemination than with local tumour growth alone. Previous studies demonstrated that glycosylation-dependent modulation of adhesion molecules and extracellular interactions may have been implicated in metastatic behaviour [18,22].

It should be emphasised that advanced pathological stage and lymph node metastasis are not independent clinical endpoints in colon cancer. According to the AJCC TNM system, Stage III disease is defined primarily by regional lymph node involvement; therefore, the association between B3GNT3 expression and advanced stage partially reflects its association with nodal status. For this reason, the present results are interpreted as evidence that B3GNT3 expression is associated with an interrelated clinicopathological pattern involving stage grouping and nodal status, rather than as evidence of two separate biological effects.

The ROC and logistic regression analyses further supported these clinicopathological associations within the analysed cohort. B3GNT3 expression demonstrated moderate discriminatory performance for identifying advanced-stage disease and lymph node metastasis. In adjusted exploratory logistic regression models, reduced B3GNT3 expression remained associated with advanced stage and nodal status after adjustment for selected clinicopathological variables. However, these models are exploratory and should not be interpreted as establishing clinical utility. In particular, the advanced-stage and nodal-status models should be regarded as complementary analyses of related outcomes, since nodal involvement is embedded within AJCC stage classification.

These regression findings must also be interpreted cautiously because of the limited number of events relative to the number of predictors. The events-per-variable ratios were 8.3 for the advanced-stage endpoint and 8.0 for the lymph-node-metastasis endpoint, both below the conventional threshold of 10. In addition, Stage IV cases were few (n = 7), and several odds ratios had wide confidence intervals, indicating limited precision. Although multicollinearity was low and calibration tests did not show a significant lack of fit, bootstrap optimism correction and repeated cross-validation demonstrated reduced model performance compared with the apparent estimates. These findings support the exploratory nature of the multivariable analyses and argue against overinterpretation of individual odds ratios.

A particularly important aspect of the present study was the exploratory evaluation of whether B3GNT3 expression contributed to model fit beyond standard pathological assessment. The inclusion of B3GNT3 expression improved the fit of models containing tumour invasion depth alone and increased pseudo-R^2^ values more than threefold. These findings suggest that B3GNT3 expression may capture cohort-level clinicopathological information not fully reflected by pT classification alone. However, this comparison was limited to pT-only and pT-plus-B3GNT3 models and was not intended to demonstrate independence from nodal status or from the AJCC stage system as a whole. Therefore, these results should be interpreted as exploratory evidence of additional clinicopathological association within this cohort, rather than as evidence of clinical predictive utility.

Western blot validation further strengthened the reliability of the immunohistochemical findings by independently confirming increased B3GNT3 protein expression in tumour tissue. In addition, immunogold labelling by the use of TEM demonstrated predominant localisation of B3GNT3 within the Golgi apparatus and secretory pathway compartments, which is fully consistent with the known intracellular localisation and biological role of glycosyltransferases. These findings support the biological plausibility of the observed protein-level alterations and further indicate active involvement of B3GNT3 in tumour-associated glycosylation processes. These findings should be interpreted with particular caution. This component of the study was included to provide supportive information on the subcellular localisation of B3GNT3 immunoreactivity and was not designed as a quantitative analysis of protein abundance. Although immunogold labelling was observed predominantly in intracellular compartments consistent with the Golgi apparatus and secretory pathway, no particle-density quantification was performed. 

The relationship between B3GNT3 expression and tumour immunity remains incompletely understood. Previous studies suggested associations between glycosylation-related pathways and modulation of immune responses within the tumour microenvironment [23]. This discrepancy may again reflect tissue-specific differences in glycosylation-dependent immune regulation or indicate that B3GNT3 influences immune function through mechanisms not directly reflected by the quantity of inflammatory infiltrates.

Several limitations of the present study should be acknowledged. First, the retrospective single-centre design and relatively limited cohort size may restrict the generalisability of the findings. Because the cohort was based on available archival tissue material, no prospective sample-size calculation was performed before patient inclusion. Although the available sample size was considered sufficient to detect the main observed IHC-based differences, it remains limited for subgroup analyses, ROC-derived cut-off validation, and multivariable prediction modelling. Second, survival, recurrence, and treatment-response data were not available, precluding direct evaluation of the prognostic or predictive significance of B3GNT3 expression. Therefore, the present results demonstrate associations with clinicopathological features only and should not be interpreted as evidence of prognostic, predictive, or clinically validated biomarker utility. Third, advanced stage and lymph node metastasis are interrelated clinicopathological endpoints rather than independent biological outcomes, because AJCC stage III disease is largely defined by nodal positivity. Accordingly, the stage-based analyses partly overlap with the nodal-status analyses and should be interpreted as related clinicopathological associations within this retrospective cohort.

Another limitation concerns the exploratory ROC-derived cut-off. The threshold of IRS ≤ 8 was selected using the Youden index and evaluated in the same retrospective dataset. This approach may overestimate performance metrics, particularly in a relatively small cohort without an independent validation set. Although sensitivity, specificity, PPV, NPV, and their confidence intervals are now provided, the AUC values were moderate, and the cut-off should be interpreted only as an exploratory threshold requiring validation in larger external cohorts.

The multivariable logistic regression models should also be interpreted cautiously. The cohort included 97 patients, with 50 advanced-stage events and 48 node-positive events, while the full models included six predictors. The resulting events-per-variable ratios of 8.3 and 8.0 are below the conventional threshold of 10, and the small number of Stage IV cases (n = 7) further limits the stability of stage-related estimates. Although additional diagnostics, calibration assessment, bootstrap resampling, and repeated cross-validation were performed, the regression results should be considered exploratory and hypothesis-generating.

Finally, although Western blot and ultrastructural analyses strengthened the biological plausibility of the findings, they also have methodological limitations. Western blot analysis was performed in a limited subset of paired samples and was interpreted as supportive protein-level validation. Immunogold analysis was descriptive rather than quantitative, and no particle-density quantification or statistical comparison of immunogold labelling intensity was performed. Functional mechanistic studies were also not performed, and molecular stratification according to MSI and KRAS/BRAF status was beyond the scope of the present study. Future investigations should therefore include independent validation cohorts, survival and treatment-response analyses, and mechanistic studies evaluating the role of B3GNT3 in glycosylation remodelling and colorectal cancer biology.

In conclusion, the present study demonstrates that B3GNT3 expression is altered in colon adenocarcinoma and shows a complex association with clinicopathological features. Increased B3GNT3 protein expression was observed in tumour tissue compared with non-neoplastic mucosa and in poorly differentiated lesions, whereas reduced B3GNT3 expression was associated with advanced stage and nodal status in adjusted exploratory models.

These findings differ from observations reported in several other malignancies, highlighting the context-dependent and tumour-specific role of B3GNT3 in cancer biology. However, because disease-free survival, overall survival, recurrence, treatment-response data, and an external validation cohort were not available, the present study does not establish B3GNT3 as a prognostic, predictive, or clinically useful biomarker. Advanced stage and lymph node metastasis should be viewed as interrelated clinicopathological features, and the observed associations should not be interpreted as independent biological endpoints. The multivariable regression analyses should also be interpreted cautiously because of the limited events-per-variable ratios, wide confidence intervals, and modest optimism observed during internal validation. The ROC-derived cut-off of IRS ≤8 remains exploratory and should not be used as a validated clinical threshold without independent external validation.

## 4. Materials and Methods

### 4.1. Bioinformatic Analysis of B3GNT3 Expression in Public Datasets

#### 4.1.1. TCGA Transcriptomic Analysis

Transcriptomic data for colon adenocarcinoma (COAD) were obtained from The Cancer Genome Atlas (TCGA) project via the UALCAN interactive web platform (http://ualcan.path.uab.edu).

B3GNT3 mRNA expression levels were analysed in primary colon adenocarcinoma tissues and normal colonic samples. Gene expression values were presented as transcripts per million (TPM). Tumour samples were stratified according to pathological stage (Stage I–IV), patient sex, and lymph node status (N0, N1, N2), as defined in the TCGA clinical annotations.

Pairwise comparisons between groups were performed using statistical tests implemented within the UALCAN platform. A *p*-value < 0.05 was considered statistically significant.

#### 4.1.2. CPTAC Proteomic Analysis

Protein expression data were obtained from the Clinical Proteomic Tumour Analysis Consortium (CPTAC) colon cancer cohort via the UALCAN platform. Protein abundance values were expressed as Z-scores normalised relative to normal tissue samples.

B3GNT3 protein levels were compared between normal colonic tissues and tumours stratified by pathological stage (Stage I–IV) and patient sex. Statistical significance was determined using platform-embedded statistical methods.

### 4.2. Immunohistochemistry

The study included 97 patients with histologically confirmed colon adenocarcinoma who underwent surgical resection. Archival anonymised formalin-fixed, paraffin-embedded (FFPE) tissue blocks were retrieved from the institutional archive.

Clinicopathological data were collected retrospectively and included: age at diagnosis, sex, tumour location (left vs. right colon), histological grade (G1–G3), depth of invasion (pT), regional lymph node status (pN), pathological stage according to the American Joint Committee on Cancer (AJCC) TNM classification, 8th edition. For statistical analysis, tumours were categorised as early stage (I–II) or advanced stage (III–IV).

Archival paraffin-embedded tissue blocks of colon adenocarcinoma (COAD) and corresponding normal colonic mucosa, obtained from a macroscopically unchanged area of the resected specimen located at least 5 cm from the tumour and confirmed to be free of neoplastic or dysplastic lesions, were retrieved from the archive.

Sections (4 µm thick) were cut from the blocks and mounted on Polysine slides. The sections were deparaffinized in xylene and rehydrated through a graded series of ethanol.

Antigen retrieval was performed by microwave heating in 10 mM citrate buffer (pH 6.0) for 2 × 8 min. After cooling and washing, the sections were incubated with rabbit anti-B3GNT3 antibody (GeneTex, Irvine, CA, USA; GTX108928) at a dilution of 1:600 for 24 h at 4 °C. Protein expression was visualised using the BrightVision detection system (WellMed BV, Arnhem, The Netherlands; Cat. No. DPVB55HRP) and Permanent AP Red Chromogen (Agilent Technologies, Santa Clara, CA, USA; Dako Code K0640). Nuclei were counterstained with Mayer’s haematoxylin. 

Immunohistochemical staining was evaluated using the immunoreactive score (IRS), based on staining intensity and the percentage of positive cells. Staining intensity was graded as follows: 0 (no signal), 1 (weak), 2 (moderate), and 3 (strong). The percentage of positive cells was scored as follows: 0 (negative), 1 (10–25%), 2 (26–50%), 3 (51–75%), and 4 (76–100%). The final score (0–12) was obtained by multiplying intensity and percentage values. The IHC analysis included 97 colon adenocarcinoma samples. Adjacent non-neoplastic colonic mucosa was evaluated where available and analysed separately from tumour tissue. The final consensus IRS values were treated as semi-quantitative numerical scores for statistical analysis.

Immunohistochemical scoring was performed on whole-tissue sections. For each case, the entire slide was first reviewed at low magnification, and three representative viable areas of invasive tumour were selected from each slide for IRS assessment. Only unequivocal cytoplasmic and/or apical cytoplasmic staining in viable neoplastic epithelial cells was considered positive. Stromal staining, inflammatory cells, necrotic areas, mucin pools, luminal debris, tissue folds, edge artefacts, and nonspecific background staining were excluded from the tumour-cell IRS evaluation. In cases with heterogeneous staining, scoring was based on the predominant staining pattern within viable invasive tumour tissue, while areas showing markedly different staining intensity were additionally reviewed to avoid selection bias. Adjacent non-neoplastic colonic mucosa was evaluated separately as a reference tissue compartment and was not included in the tumour IRS.

The slides were evaluated independently by two observers (M.B.-Z. and A.P.) experienced in histological and immunohistochemical assessment, and the observers were blinded to clinicopathological data at the time of scoring. Interobserver reproducibility was assessed before consensus scoring by comparing independently assigned IRS values. Cases with discrepant scores were re-evaluated jointly, and a final consensus IRS was assigned for statistical analysis.

Negative-control IHC sections were processed in parallel by omitting the primary antibody while retaining the same detection system and chromogen. Representative negative-control IHC images were added to the Supplementary Material to document the absence of relevant nonspecific staining.

### 4.3. Western Blot Analysis

Protein extraction was performed using a lysis buffer containing 20 mM Tris–HCl, 150 mM NaCl, 2 mM EDTA, 1% Triton X–100, 10% glycerol, and 1 mM PMSF (pH 7.4), supplemented with protease and phosphatase inhibitor cocktails (Protease Inhibitor Cocktail, Carl Roth GmbH + Co. KG, Karlsruhe, Germany; PhosphoSTOP, Roche Diagnostics, Basel, Switzerland).

Tissue samples (1 mg) were incubated with 20 µL of lysis buffer on ice for 15 min, followed by mechanical homogenization (TissueRuptor, QIAGEN, Hilden, Germany) using short pulses and subsequent ultrasonication on ice (Vibra–Cell™ Ultrasonic Liquid Processor, Sonics & Materials, Inc., Newtown, CT, USA) in 15 s pulses with 10 s intervals. Homogenates were incubated on ice for an additional 30 min and centrifuged at 12,000 rpm for 10 min at 4 °C. The supernatants were collected and stored at −80 °C until further analysis.

Total protein was extracted from tissue samples, and protein concentration was determined using a colourimetric assay. Equal amounts of protein (50 µg per lane) were separated by 10% SDS–polyacrylamide gel electrophoresis (SDS-PAGE) for 1.5 h at 150 V and subsequently transferred onto nitrocellulose membranes by wet transfer in transfer buffer for 2 h at 300 mA at 4 °C.

Membranes were blocked with 5% skim milk in TBST for 30 min at room temperature and then incubated overnight at 4 °C with rabbit anti-B3GNT3 primary antibody (GeneTex, GTX108928; 1:4000) diluted in blocking solution. After washing with TBST, membranes were incubated for 1 h at room temperature with HRP-conjugated mouse anti-rabbit IgG secondary antibody (Invitrogen, Carlsbad, CA, USA, 31464; 1:25,000) diluted in 5% skim milk/TBST.

Protein bands were visualised using SuperSignal^®^ West Femto Maximum Sensitivity Substrate (Thermo Scientific, Waltham, MA, USA) and exposed to Amersham Hyperfilm ECL (GE Healthcare, Chicago, IL, USA).

Densitometric analysis was performed using ImageJ software (version 1.54d, National Institutes of Health, Bethesda, MD, USA), with β-actin serving as the loading control. Although tumour and paired control samples were resolved on separate gels due to the total number of samples (13 paired specimens, 26 lanes), all membranes were processed under identical conditions, including blocking, primary and secondary antibody incubation, and chemiluminescent substrate application. All membranes were exposed simultaneously to the same film for 10 s, ensuring comparable signal detection across tumour and control samples.

### 4.4. Immunogold Transmission Electron Microscopy

For ultrastructural localisation of B3GNT3, tissue samples were fixed in 4% paraformaldehyde in 0.1 M PBS for 2 h at room temperature, rinsed in PBS, dehydrated in graded ethanol, and embedded in LR White resin. Ultrathin sections (70 nm) were cut using an RMC Boeckeler PowerTome PC ultramicrotome with a diamond knife (Diatom AG, Hvidovre, Denmark) and mounted on Formvar-coated nickel grids.

Sections were pre-incubated in 50 mM NH_4_Cl (30 min) and blocked with 1% BSA in PBS (30 min). Grids were then incubated overnight at 4 °C with rabbit anti-B3GNT3 antibody (GeneTex; GTX108928; 1:4000). Immunogold labelling was performed using 15 nm gold-conjugated goat anti-rabbit IgG (BBInternational BBI Solutions, Crumlin, UK; 1:100) for 1 h.

After washing, sections were contrasted with 0.5% uranyl acetate. Negative controls were processed without the primary antibody.

Grids were examined using a TECNAI 12 G2 Spirit BioTwin transmission electron microscope (FEI Company, Hillsboro, OR, USA) at 120 kV, and images were captured with a Morada CCD camera (Gatan, Pleasanton, CA, USA).

### 4.5. Statistical Analysis

Statistical analyses were performed using Statistica ver. 9.1 and PQStat ver. 1.8.2. Continuous variables were expressed as mean ± standard deviation (SD) or median with interquartile range (Q1–Q3), as appropriate. Group comparisons were performed using the Mann–Whitney U test for two-group comparisons and the Kruskal–Wallis test with post hoc analysis for multiple-group comparisons. Correlations were assessed using Spearman’s rank correlation coefficient (R).

Receiver operating characteristic (ROC) curve analysis was used as an exploratory assessment of the association between B3GNT3 expression and advanced stage (III/IV vs. I/II) or lymph node metastasis (pN1–2 vs. pN0). Area under the curve (AUC), 95% confidence intervals (CI), sensitivity, specificity, positive predictive value (PPV), negative predictive value (NPV), accuracy, and optimal cut-off values based on the Youden index were calculated. Confidence intervals for sensitivity, specificity, PPV, NPV, and accuracy were calculated using Wilson’s score intervals. Because the ROC-derived cut-off was identified and evaluated in the same dataset, it was treated as exploratory and was not considered an externally validated clinical threshold.

Logistic regression analysis was performed to identify factors associated with advanced pathological stage and lymph node metastasis. Because AJCC stage III colon cancer is defined primarily by lymph node involvement, advanced pathological stage, and lymph node metastasis were treated as interrelated outcomes. Nodal status was not included as an adjustment variable in models using advanced stage as the outcome, to avoid circular adjustment for a component of the endpoint. The two sets of analyses were interpreted as complementary rather than independent.

Both univariate and multivariate models were constructed. Two modelling strategies were applied: B3GNT3 as a continuous variable and B3GNT3 as a dichotomous variable using the exploratory ROC-derived cut-off (IRS ≤ 8). Incremental model fit was evaluated by comparing nested logistic regression models: a reduced model including only the pathological T (pT) parameter and an extended model incorporating both pT and B3GNT3 expression. Model improvement was quantified using changes in pseudo-R^2^ and likelihood ratio tests. These analyses were exploratory and were not intended to establish prognostic or predictive clinical utility.

To address potential overfitting, events-per-variable ratios were calculated for each full multivariable model. Multicollinearity was assessed using variance inflation factors. Calibration was evaluated using the Hosmer–Lemeshow goodness-of-fit test and Brier scores. Internal validation was performed using bootstrap resampling and repeated 10-fold cross-validation, with apparent and optimism-corrected model performance reported using AUC and Brier score estimates.

Because this was a retrospective single-centre study based on available archival tissue material, no prospective sample-size calculation was performed before patient inclusion. The cohort size was determined by the number of eligible colon adenocarcinoma cases with available clinicopathological data and suitable tissue material. To address sample-size adequacy, a post hoc power/sensitivity assessment was performed for the main IHC-based comparisons. The available cohort of 97 colon adenocarcinoma cases, including 47 early-stage and 50 advanced-stage tumours as well as 49 node-negative and 48 node-positive cases, was considered sufficient to detect the main observed differences in IRS-based B3GNT3 expression between these groups. However, the cohort size remains limited for subgroup analyses, ROC-derived cut-off validation, and multivariable prediction modelling. Therefore, the statistically significant findings should be interpreted as exploratory clinicopathological associations within this retrospective cohort.

A *p*-value < 0.05 was considered statistically significant. Statistical analysis was performed by M.B.-Z. and A.P.

## Figures and Tables

**Table 1:** Clinicopathological characteristics of the study cohort (n = 97).

Variable	Category	N	%
Sex	Female	48	49.48
	Male	49	50.52
Age at diagnosis (years)	≤60	29	29.90
	61–75	33	34.02
	>75	35	36.08
	Mean ± SD	67.8 ± 12.3	—
	Median (Q1–Q3)	68 (59–78)	—
	Range	33–89	—
Pathological stage (AJCC TNM, 8th edition [3])	Stage I	10	10.31
	Stage II	37	38.14
	Stage III	43	44.33
	Stage IV	7	7.22
Histological grade	G1 (well differentiated)	14	14.43
	G2 (moderately differentiated)	59	60.82
	G3 (poorly differentiated)	24	24.74
Primary tumour (pT)	pT1	3	3.09
	pT2	10	10.31
	pT3	66	68.04
	pT4	18	18.56
Regional lymph nodes (pN)	pN0	49	50.52
	pN1	33	34.02
	pN2	15	15.46
Tumour location	Left-sided colon	54	55.67
	Right-sided colon	43	44.33

**Table 2:** Association between B3GNT3 immunoreactive score (IRS) and clinicopathological parameters in colon adenocarcinoma patients. IRS values represent the predefined semi-quantitative IHC scoring system used in this study instead of the classical H-score.

Parameter	Group	N	Median (Q1–Q3)	*p*-Value
Overall		97	9 (7–10)	–
Pathological stage	I	10	10 (8–10)	
	II	37	10 (9–11)	
	III	43	8 (6–10)	
	IV	7	6 (4–8)	*p* < 0.001
Stage (grouped)	I–II	47	10 (9–11)	
	III–IV	50	8 (6–9)	*p* < 0.001
Sex	Female	48	10 (8–11)	
	Male	49	9 (7–9)	*p* = 0.011
Age at diagnosis	≤60 years	29	9 (8–10)	
	61–75 years	33	9 (7–11)	
	>75 years	35	9 (6–10)	*p* = 0.229
Histological grade	G1	14	7.5 (6–9)	
	G2	59	9 (7–10)	
	G3	24	9.5 (8–11)	*p* = 0.048
Tumour depth (T)	T1–T2	13	10 (8–10)	
	T3–T4	84	9 (7–10)	*p* = 0.441
Lymph node status (N)	N0	49	10 (9–11)	
	N1–N2	48	8 (6–9)	*p* < 0.001
Tumour location	Left colon	54	8 (7–10)	
	Right colon	43	9 (8–11)	*p* = 0.304

**Table 3:** Exploratory ROC-derived cut-off analysis for B3GNT3 expression in relation to advanced stage and lymph node metastasis. The IRS cut-off of 8 was identified and evaluated in the same retrospective cohort and should therefore be interpreted as exploratory. Confidence intervals for sensitivity, specificity, PPV, and NPV were calculated using Wilson score intervals.

Outcome	AUC (95% CI)	Exploratory Cut-Off	TP/FP/TN/FN	Sensitivity (95% CI)	Specificity (95% CI)	PPV (95% CI)	NPV (95% CI)
Stage III–IV vs. I–II	0.740 (0.636–0.843)	IRS ≤ 8	32/9/38/18	64.00% (50.14–75.86)	80.85% (67.46–89.58)	78.05% (63.29–88.00)	67.86% (54.82–78.60)
pN1–2 vs. pN0	0.706 (0.597–0.814)	IRS ≤ 8	30/11/38/18	62.50% (48.36–74.78)	77.55% (64.12–86.98)	73.17% (58.07–84.31)	67.86% (54.82–78.60)

**Table 4:** Logistic regression analysis for advanced stage (III/IV)—B3GNT3 as a continuous variable. Univariate and multivariate models.

Variable	Univariate OR	*p*	95% CI	Multivariate OR	*p*	95% CI
Sex, M (reference: Female)	1.576	0.266	0.707–3.512	0.965	0.941	0.371–2.509
Age	1.028	0.113	0.994–1.063	1.016	0.409	0.978–1.055
B3GNT3	0.608	<0.001	0.474–0.779	0.607	<0.001	0.464–0.794
G3 (reference: G1/G2)	0.921	0.861	0.366–2.317	1.307	0.631	0.438–3.896
T3/T4 (reference: T1/T2)	4.234	0.038	1.086–16.501	4.760	0.054	0.973–23.296
Tumour location P (reference: L)	0.973	0.946	0.436–2.168	0.960	0.933	0.372–2.480

**Table 5:** Logistic regression analysis for advanced stage (III/IV)—B3GNT3 as a dichotomous variable (cut-off 8). Univariate and multivariate models.

Variable	Univariate OR	*p*	95% CI	Multivariate OR	*p*	95% CI
Sex, M (reference: Female)	1.576	0.266	0.707–3.512	1.195	0.718	0.454–3.142
Age	1.028	0.113	0.994–1.063	1.021	0.302	0.982–1.061
B3GNT3 (≤8 vs. >8)	7.506	<0.001	2.967–18.988	8.605	<0.001	3.001–24.673
G3 (reference: G1/G2)	0.921	0.861	0.366–2.317	1.247	0.700	0.406–3.831
T3/T4 (reference: T1/T2)	4.234	0.038	1.086–16.501	5.320	0.041	1.073–26.371
Tumour location P (reference: L)	0.973	0.946	0.436–2.168	1.350	0.553	0.501–3.635

**Table 6:** Comparison of logistic regression models for advanced stage (III/IV)—B3GNT3 as a continuous variable. Extended model (T + B3GNT3) vs. reduced model (T only).

Variable	Model 1: Extended OR	*p*	95% CI	Model 2: Reduced OR	*p*	95% CI
B3GNT3	0.603	<0.001	0.468–0.779	–	–	–
T3/T4 (reference: T1/T2)	4.760	0.049	1.004–21.247	4.234	0.038	1.086–16.501
Pseudo R^2^	0.188	–	–	0.038	–	–
Model comparison	*p* < 0.001	–	–	–	–	–

**Table 7:** Comparison of logistic regression models for advanced stage (III/IV)—B3GNT3 as a dichotomous variable (cut-off value of 8). Extended model (T + B3GNT3) vs. reduced model (T only).

Variable	Model 1: Extended OR	*p*	95% CI	Model 2: Reduced OR	*p*	95% CI
B3GNT3 (≤8 vs. >8)	8.420	<0.001	3.144–22.550	–	–	–
T3/T4 (reference: T1/T2)	5.662	0.027	1.215–26.391	4.234	0.038	1.086–16.501
Pseudo R^2^	0.198	–	–	0.038	–	–
Model comparison	*p* < 0.001	–	–	–	–	–

**Table 8:** Logistic regression analysis for lymph node metastasis (N+)—B3GNT3 as a continuous variable. Univariate and multivariate models.

Variable	Univariate OR	*p*	95% CI	Multivariate OR	*p*	95% CI
Sex, M (reference: Female)	1.578	0.265	0.708–3.516	1.055	0.911	0.411–2.705
Age	1.022	0.193	0.989–1.057	1.012	0.536	0.975–1.050
B3GNT3	0.677	<0.001	0.542–0.846	0.680	0.002	0.532–0.868
G3 (reference: G1/G2)	1.028	0.954	0.409–2.585	1.379	0.558	0.471–4.043
T3/T4 (reference: T1/T2)	6.658	0.018	1.390–31.897	7.868	0.017	1.437–43.098
Tumour location P (reference: L)	0.807	0.601	0.362–1.802	0.682	0.413	0.272–1.705

Abbreviations: OR, odds ratio; CI, confidence interval; T, tumour depth; B3GNT3, beta-1,3-N-acetylglucosaminyltransferase 3.

**Table 9:** Logistic regression analysis for lymph node metastasis (N+)—B3GNT3 as a dichotomous variable (cut-off value of 8). Univariate and multivariate models.

Variable	Univariate OR	*p*	95% CI	Multivariate OR	*p*	95% CI
Sex, M (reference: Female)	1.578	0.265	0.708–3.516	1.239	0.659	0.478–3.216
Age	1.022	0.193	0.989–1.057	1.015	0.435	0.977–1.055
B3GNT3 (≤8 vs. >8)	5.758	<0.001	2.365–14.018	6.216	<0.001	2.262–17.081
G3 (reference: G1/G2)	1.028	0.954	0.409–2.585	1.343	0.601	0.444–4.058
T3/T4 (reference: T1/T2)	6.658	0.018	1.390–31.897	8.769	0.014	1.551–49.565
Tumour location P (reference: L)	0.807	0.601	0.362–1.802	0.912	0.851	0.349–2.380

Abbreviations: OR, odds ratio; CI, confidence interval; T, tumour depth; B3GNT3, beta-1,3-N-acetylglucosaminyltransferase 3.

**Table 10:** Logistic regression analysis for lymph node metastasis (N+)—B3GNT3 as a continuous variable. Comparison of extended and reduced models.

Variable	Model 1: Extended (B3GNT3 + T) OR	*p*	95% CI	Model 2: Reduced (T only) OR	*p*	95% CI
B3GNT3	0.672	<0.001	0.533–0.847	–	–	–
T3/T4 (reference: T1/T2)	7.249	0.021	1.352–38.864	6.658	0.018	1.389–31.897
Pseudo R^2^	0.184	–	–	0.057	–	–
Model comparison	*p* < 0.001	–	–	–	–	–

Abbreviations: OR, odds ratio; CI, confidence interval; T, tumour depth; B3GNT3, beta-1,3-N-acetylglucosaminyltransferase 3.

## Data Availability

The datasets generated and analysed during the current study are available from the corresponding author upon reasonable request.

## Supplementary Materials

The following supporting information can be downloaded at: https://www.mdpi.com/article/10.3390/ijms27146273/s1.

## References

[1] Sung H., Ferlay J., Siegel R.L., Laversanne M., Soerjomataram I., Jemal A., Bray F. (2021). Global Cancer Statistics 2020: GLO-BOCAN Estimates of Incidence and Mortality Worldwide for 36 Cancers in 185 Countries. CA Cancer J. Clin..

[2] Siegel R.L., Giaquinto A.N., Jemal A. (2024). Cancer statistics, 2024. CA Cancer J. Clin..

[3] Amin M.B., Greene F.L., Edge S.B., Compton C.C., Gershenwald J.E., Brookland R.K., Meyer L., Gress D.M., Byrd D.R., Winchester D.P. (2017). The Eighth Edition AJCC Cancer Staging Manual: Continuing to build a bridge from a population-based to a more “personalized” approach to cancer staging. CA Cancer J. Clin..

[4] Dienstmann R., Salazar R., Tabernero J. (2015). Personalizing colon cancer adjuvant therapy: Selecting optimal treatments for individual patients. J. Clin. Oncol..

[5] Guinney J., Dienstmann R., Wang X., de Reyniès A., Schlicker A., Soneson C., Marisa L., Roepman P., Nyamundanda G., Angelino P. (2015). The consensus molecular subtypes of colorectal cancer. Nat. Med..

[6] Ramos-Vara J.A., Miller M.A. (2014). When tissue antigens and antibodies get along: Revisiting the technical aspects of immuno-histochemistry—The red, brown, and blue technique. Vet. Pathol..

[7] Tan W.C.C., Nerurkar S.N., Cai H.Y., Ng H.H.M., Wu D., Wee Y.T.F., Lim J.C.T., Yeong J., Lim T.K.H. (2020). Overview of multiplex immunohistochemistry/immunofluorescence techniques in the era of cancer immunotherapy. Cancer Commun..

[8] Fedchenko N., Reifenrath J. (2014). Different approaches for interpretation and reporting of immunohistochemistry analysis results in the bone tissue—A review. Diagn. Pathol..

[9] Pinho S.S., Reis C.A. (2015). Glycosylation in cancer: Mechanisms and clinical implications. Nat. Rev. Cancer.

[10] Munkley J., Elliott D.J. (2016). Hallmarks of glycosylation in cancer. Oncotarget.

[11] Varki A. (2017). Biological roles of glycans. Glycobiology.

[12] Togayachi A., Kozono Y., Ishida H., Abe S., Suzuki N., Tsunoda Y., Hagiwara K., Kuno A., Ohkura T., Sato N. (2007). Polylactosamine on glycoproteins influences basal levels of lymphocyte and macrophage activation. Proc. Natl. Acad. Sci. USA.

[13] Zhuang H., Zhou Z., Zhang Z., Chen X., Ma Z., Huang S., Gong Y., Zhang C., Hou B. (2020). B3GNT3 overexpression promotes tumor progression and inhibits infiltration of CD8+ T cells in pancreatic cancer. Aging.

[14] Gao L., Zhang H., Zhang B., Zhu J., Chen C., Liu W. (2018). B3GNT3 overexpression is associated with unfavourable survival in non-small cell lung cancer. J. Clin. Pathol..

[15] Wu Y., Luo J., Li H., Huang Y., Zhu Y., Chen Q. (2022). B3GNT3 as a prognostic biomarker and correlation with immune cell infiltration in lung adenocarcinoma. Ann. Transl. Med..

[16] Liu Y., Beyer A., Aebersold R. (2016). On the dependency of cellular protein levels on mRNA abundance. Cell.

[17] Vogel C., Marcotte E.M. (2012). Insights into the regulation of protein abundance from proteomic and transcriptomic analyses. Nat. Rev. Genet..

[18] Ho W.L., Che M.I., Chou C.H., Chang H.H., Jeng Y.M., Hsu W.M., Lin K.H., Huang M.C. (2013). B3GNT3 expression suppresses cell migration and invasion and predicts favorable outcomes in neuroblastoma. Cancer Sci..

[19] Zhang W., Hou T., Niu C., Song L., Zhang Y. (2015). B3GNT3 expression is a novel marker correlated with pelvic lymph node metastasis and poor clinical outcome in early-stage cervical cancer. PLoS ONE.

[20] Sun Y., Liu T., Xian L., Liu W., Liu J., Zhou H. (2020). B3GNT3, a direct target of miR-149-5p, promotes lung cancer development and indicates poor prognosis of lung cancer. Cancer Manag. Res..

[21] Yamashita K., Kuno A., Matsuda A., Ikehata Y., Katada N., Hirabayashi J., Narimatsu H., Watanabe M. (2016). Lectin microarray technology identifies specific lectins related to lymph node metastasis of advanced gastric cancer. Gastric Cancer.

[22] Pereira M.S., Alves I., Vicente M., Campar A., Silva M.C., Padrão N.A., Pinto V., Fernandes Â., Dias A.M., Pinho S.S. (2018). Glycans as key checkpoints of T cell activity and function. Front. Immunol..

[23] Rodrigues E., Macauley M.S. (2018). Hypersialylation in cancer: Modulation of inflammation and therapeutic opportunities. Cancers.

