# Supplementary material for: Association of B3GNT3 Expression with Tumour Stage and Lymph Node Metastasis in Colon Adenocarcinoma
Source: Int J Mol Sci. 2026 Jul 14;27(14):6273. doi: 10.3390/ijms27146273 (PMC13409940; doi:10.3390/ijms27146273)
Supplement: Supplementary file 1 [file ijms-27-06273-s001.zip › ijms-4357133-supplementary.pdf]

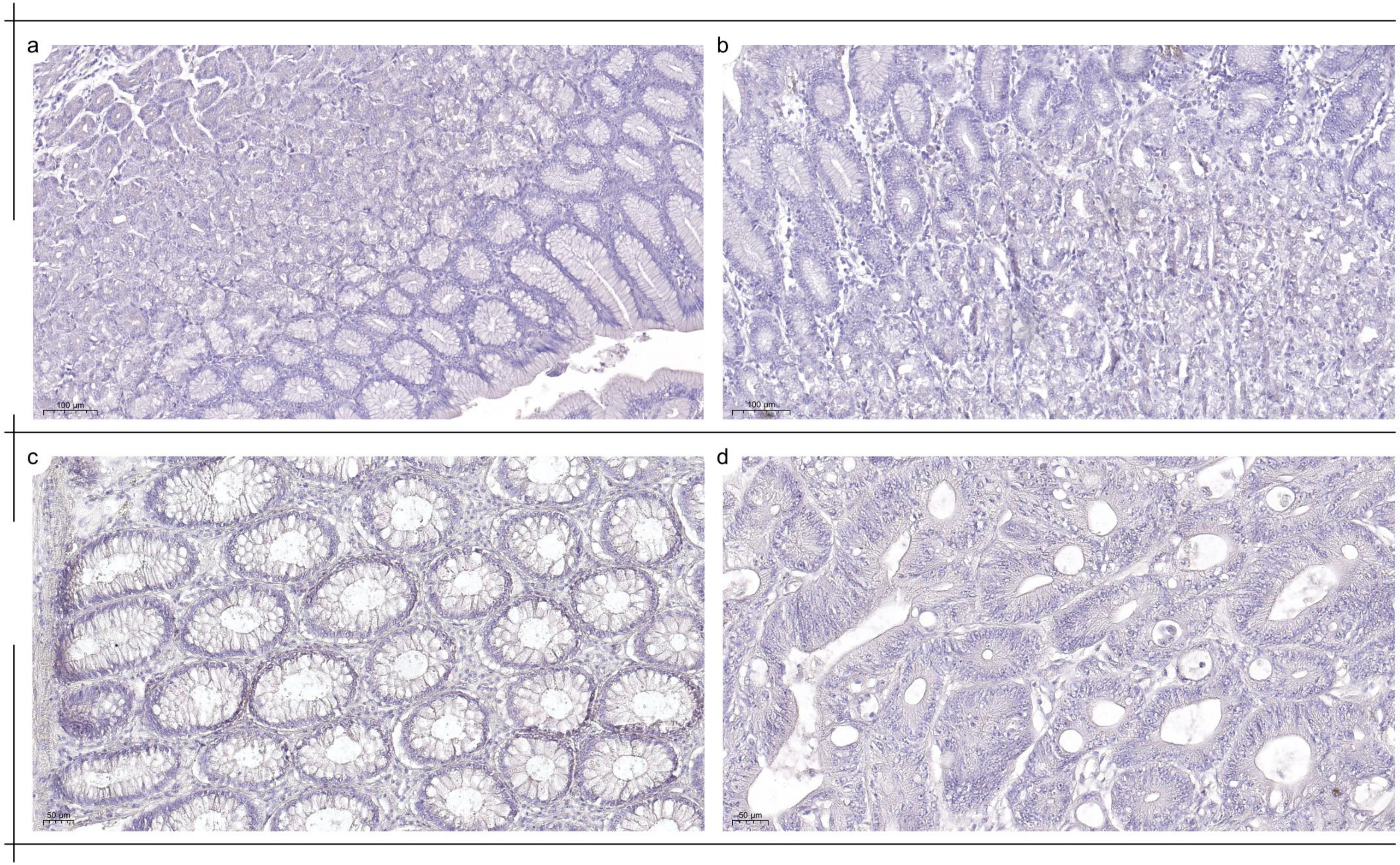

Figure S1. Representative negative-control immunohistochemical staining. Negative-control sections were processed by omission of the primary anti-B3GNT3 antibody while retaining the same detection system and chromogen. No relevant specific red immunoreactivity was observed. Panels c and d show non-neoplastic colonic mucosa, which was used as the tissue-relevant negative control for the present colon adenocarcinoma study. In addition, non-neoplastic gastric mucosa was included as an additional gastrointestinal tissue control to assess the absence of nonspecific staining in another non-neoplastic mucosal tissue compartment (a,b). Nuclei were counterstained with haematoxylin and appear blue-purple. Scale bars: 100 µm (a,b) and 50 µm (c,d).
